# GapFiller: a de novo assembly approach to fill the gap within paired reads

**DOI:** 10.1186/1471-2105-13-S14-S8

**Published:** 2012-09-07

**Authors:** Francesca Nadalin, Francesco Vezzi, Alberto Policriti

**Affiliations:** 1 Department of Mathematics and Computer Science, University of Udine, Udine 33100, Italy; 2 IGA Applied Genomics Institute, Udine 33100, Italy; 3 Science for Life Laboratory, KTH Royal Institute of Technology, Solna 17121, Sweden

**Keywords:** *de novo *assembly, paired reads, (Hamming-aware) hash functions, Next Generation Sequencing data

## Abstract

**Background:**

Next Generation Sequencing technologies are able to provide high genome coverages at a relatively low cost. However, due to limited reads' length (from 30 bp up to 200 bp), specific bioinformatics problems have become even more difficult to solve. *De novo *assembly with short reads, for example, is more complicated at least for two reasons: first, the overall amount of "noisy" data to cope with increased and, second, as the reads' length decreases the number of *unsolvable *repeats grows. Our work's aim is to go at the root of the problem by providing a pre-processing tool capable to produce (in-silico) longer and highly accurate sequences from a collection of Next Generation Sequencing reads.

**Results:**

In this paper a seed-and-extend *local *assembler is presented. The kernel algorithm is a loop that, starting from a read used as seed, keeps extending it using heuristics whose main goal is to produce a collection of error-free and longer sequences. In particular, GapFiller carefully detects reliable overlaps and operates clustering similar reads in order to reconstruct the missing part between the two ends of the same insert. Our tool's output has been validated on 24 experiments using both simulated and real paired reads datasets. The output sequences are declared correct when the seed-mate is found. In the experiments performed, GapFiller was able to extend high percentages of the processed seeds and find their mates, with a false positives rate that turned out to be nearly negligible.

**Conclusions:**

GapFiller, starting from a sufficiently high short reads coverage, is able to produce high coverages of accurate longer sequences (from 300 bp up to 3500 bp). The procedure to perform safe extensions, together with the mate-found check, turned out to be a powerful criterion to guarantee contigs' correctness. GapFiller has further potential, as it could be applied in a number of different scenarios, including the post-processing validation of insertions/deletions detection pipelines, pre-processing routines on datasets for *de novo *assembly pipelines, or in any hierarchical approach designed to assemble, analyse or validate *pools *of sequences.

## Background

The recent Next Generation Sequencing (NGS) breakthrough and the consequent tremendous increase in data production, have been accompanied by the appearance of a multitude of pipelines able to *assemble *the (relatively) short sequences (*i.e*. reads) produced by state-of-the-art sequencers.

In the last two years more than 20 new *assemblers *(see [1] for an up-to-date overview) have been proposed, more than doubling in size the population of the assemblers designed for *long *Sanger reads. Despite the practical and theoretical problems involved in assembling complex genomes using only short sequences [2], several *de novo *assembly projects based exclusively on NGS data have started. Among the most popular ones we mention the Panda genome project [3], the assembly of specific human Individuals [4] (Han Chinese and Yoruban), and several other species [5].

While several tools became publicly available and several projects based on such tools started to appear, a very lively discussion on how to validate new assemblies and, in general, on how to estimate assemblers' output started. As noticed in [6], all assembly tools are based on a small number of algorithms and differ from one another only in matter of details that, very often, relate to how they deal with errors, inconsistencies, and ambiguities. As a consequence, an increasing number of studies is now being published aiming, on the one hand, at evaluating *de novo *assemblers and assemblies, and, on the other hand, at criticising the results achieved so far.

Assemblathon [7] first and second editions, dnGASP [8], and GAGE [9] try to assess the performances of existing tools triggering an assembly evaluation competition among several bioinformatics groups. Even though these competitions succeeded in giving a fairly complete overview of the assemblers' potentialities, they are almost always based on specific (often already sequenced) genomes or on simulated data, leaving open the question of whether the same tools would have had the same performances when run on different datasets (*i.e*., different genomes or real reads).

Recently proposed assemblies carried out using NGS data only (usually Illumina reads), are at the center of a lively debate. Alkan in [10] criticised two of the major late NGS achievements: the assembly of the Han Chinese and Yoruban individuals [4], both sequenced with Illumina reads. For example, Alkan identified 420.2 Mbp of missing repeated sequences from the Yoruban assembly and estimated that in both assemblies almost 16% of the genome was missing.

Some studies started to criticise the way in which the evaluation of assemblies and assemblers is carried out: standard statistics like the mean contig length and the N50 emphasize only length and nothing, or almost nothing, is said about contigs' correctness [11,12]. Evaluations of simulated data are inherently biased by the capabilities of the read simulator to faithfully reproduce error schemata [12].

More than three years after the so-called NGS revolution started, it is extremely clear that *de novo *assembly needs extensive and standardized validation steps. NGS breakthrough allowed to sequence a number of new species and individuals thought to be impossible only few years ago. While, on the one hand, an increasing number of people keeps sequencing and assemblying genomes using available assemblers and short reads, on the other one, day after day, a larger community criticises and casts doubts on assembly achievements.

At the peak of this difficult moment we try to go back to basics and propose a new tool, dubbed *GapFiller *[13], able to generate small but correct and certified contigs, that can be used either in a first step of an assembly project, or in numerous downstream analyses strongly depending on sequencing and aligning. The innovative feature of GapFiller is the possibility to produce a highly reliable output that, having been certified correct--and hence needing no further validation--, can be used, for example, to improve or validate a whole genome assembly.

Our method is based on a *seed-and-extend *schema aimed at *closing the gap *between the two mates of a paired read. Similarly to other seed-and-extend-based tools like SSAKE [14], SHARCGS [15], QSRA [16], and TAIPAN [17], GapFiller selects one read and tries to extend it using reads that overlap for a significant region. The main drawback of seed-and-extend assemblers is their inherent incapability to cope with complex (*i.e.*, repetitive) genomes. GapFiller does not aim at producing a *de novo *assembly, but only concentrates on closing the gap within paired reads. The advantages of our method lie in the generation of correct and certified contigs and, as a by-product, in the identification of "difficult" areas (*e.g.*, repeats, low covered regions, *etc*.), thus avoiding the production of wrong contigs. The assembler TAIPAN [17] is implemented to stop its extension phase in presence of a repeat; however, like all other full-fledged assemblers, it is not designed to return certified contigs as output.

Closing the gap within paired reads is a strategy already used by software packages like SHERA [18] and FLASH [19]. However, these tools are able to work only with "overlapping libraries", that is, libraries whose fragment size is shorter than twice the reads' length. GapFiller solves a more challenging problem, aiming at producing filled paired reads of higher length.

We will show how the contigs produced by our method, despite being of Sanger-like length or slightly longer (up to ~ 3500 bp), are highly reliable and correct. Moreover, the sequences produced generate a genome coverage consisting of evenly distributed long contigs. Such contigs can be used to feed another assembler (designed, for example, for long, Sanger-like, reads) or to identify and--most importantly--to reconstruct insertion and deletion events in resequencing projects.

On a more technical ground, our algorithm is based on a carefully chosen hash function together with a set of heuristics able to *avoid *or *detect *errors, as well as on a test for establishing the correctness of a sequence, that allow us to create a set of *certified *contigs.

## Methods

GapFiller is a *local *assembler based on a seed-and-extend schema [13]. Seed-and-extend assemblers repeatedly pick up a *seed *(it can be either a read or a previously assembled contig) and *extend *it using other reads. This procedure is realised by computing and analysing all--or almost all--the overlaps between seed's tips and the remaining available reads. The reads used for an *extension *are those with the highest alignment score. It is clear that the seed-and-extend assemblers' computation bottleneck is their capability to quickly cope with all the alignment scores to be determined.

GapFiller begins by storing all *useful *reads in a memory efficient data structure that allows to readily compute overlaps between the contig under construction and the remaining available reads. In a second phase each seed read (possibly belonging to a new set of paired reads) is selected one after the other and used to start an extension phase. Such phase halts when a stop condition is reached. Depending on the stop condition, the contig produced is labelled as *trusted *or *not trusted *(*i.e.*, positive or negative).

### Definitions

Let Σ be an alphabet and Σ* be the set of the words from Σ. For every *S *∈ Σ^∗ ^we will denote with |*S*| the number of characters of *S *and with *S*[*p*, . . ., *p *+ *l *- 1] the sub-sequence of *S *starting in *p *∈ {0, . . ., |*S*| - 1} and of length *l *∈ {0, . . ., |*S*| - *p*}. We will refer to *S*[*p*, . . ., *p *+ *l *- 1] as *prefix *if *p *= 0, *suffix *if *p *+ *l *= |*S*|, and as the *p-*th character of *S *if *l *= 1, and we will simply write *S*[*p*].

In order to quickly identify overlaps between the contig under construction and the reads' tips, we use an approach closely related to the one presented in [20] based on an Hamming-aware hash function. The idea is that, by representing a string of length *l *as a base-|Σ| number, one can often replace expensive char-by-char comparison by fast integer (or bit-string) comparison. However, for practical values of *l*, the integers to be compared would not fit in a memory word. For this reason, as in the classical Karp-Rabin exact string matching algorithm [21], we can work with numbers modulo *q *considering equality modulo *q *only as an indication (necessary condition) that pairs of strings may be the same (*i.e*., operating with the strings' *fingerprints*). Policriti *et al. *in [22] proposed an extension of the approach by Karp and Rabin, introducing a technique to deal with mismatches, based on the idea of replacing simple fingerprints comparison with a more articulated test. In particular they noticed that, by choosing *q *to be a Mersenne (prime, when possible) number (*i.e*., *q *= 2*^w ^*- 1, for some *w *∈ ℕ), to check whether two strings align against each other at a small Hamming distance can be implemented in average linear time.

Given a string *S *∈ Σ* and its base-|Σ| numerical representation *s *∈ ℕ, let us define the hash function $f_{H}:\sum^{*}\to \left\{0,...,q-1\right\}$ as

(1) S↦fH(S):=s modq,

where *q *is a (prime) number of the form *q *= 2*^w^*- 1, for some *w *∈ ℕ. The value *f_H _*(*S*) is called the *fingerprint *of the sequence in *S *∈ Σ* coded with *s*.

**Figure 1:**
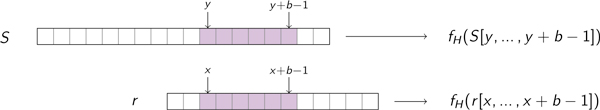
**Fingerprint computation on ***b***-length substrings**. When looking for overlaps between *S *and *r*, the fingerprints are computed on the substrings *r*[*x*, . . ., *x*+*b -*1] and *S*[*y*, . . ., *y *+ *b *- 1], respectively, where *x *and *b *are set before the contig's extension phase. We require an (almost) exact *b*-length match between *r *and *S *in order to include *r *in the set of putative overlapping reads, by setting *f_H _*(*r*[*x*, . . ., *x *+ *b *- 1]) = *f_H _*(*S*[*y*, . . ., *y *+ *b *-1]). Using such a method, the suffix-prefix overlaps that can be detected are those of length *l *≥ *x *+ *b*.

In our context, the use of *f_H _*significantly reduces the size of the set employed in the search of the overlapping reads. Every read *r*, as well as its reverse-complement, is indexed by the fingerprint of a substring of length *b*, starting at a fixed position *x *in *r *(see also Figure 1). Formally, given a set of reads ℛ, a sequence  S, a maximum allowed Hamming distance *k*, the set Z(k,q) of the *witnesses *(the Hamming sphere of radius *k *around  S, see [22] for more details), a fixed value *b *for the length of the substring on which the fingerprint is computed in *r*, and two positions *x *and *y*, the following set:

(2) $$R\left(S,x,y\right):=\left\{r\in R|\left(f_{H}\left(r\left[x,...,x+b-1\right]\right)-f_{H}\left(S\left[y,...,y+b-1\right]\right)\right)\;\;\mathrm{mod}\;q\in Z\left(k,q\right)\right\}$$

contains at least all the reads *r *∈ ℛ such that the hamming distance between *r*[*x*, . . ., *x *+ *b *- 1] and S[y,...,y+b-1] is not greater than *k*. False positives can be present but, as showed in [22], their amount is limited. On this ground the search for reads overlapping  S can be restricted to those belonging to R(S,x,y), for some x, *y *∈ ℤ.

As far as GapFiller is concerned, we set *k *= 0 as default, meaning that we search for *exact b*-length substrings in the reads (*i.e*., r[x,...,x+b-1]=S[y,...,y+b-1], for some *x *and *y*). As a consequence, better quality output will be obtained if we select a position *x *in *r *such that the average base quality is expected to be the highest possible. This point will be further discussed in the section specifically addressing data structures' design and implementation.

### Dataset preparation

In order to avoid the generation of wrong contigs, it is of utmost importance to use only correct reads over the entire extension phase. Several tools are available to perform error correction on Illumina data using the so-called "read spectrum" (consider QUAKE [23], Hammer [24], and Allpaths [25] just to mention the most recent ones). Other tools discard reads or try to improve their reliability using quality information (rNA [20] and QSRA [16]).

Our approach, when we are given raw data, is to first trim (and possibly filter) the reads on the ground of quality information using a specific rNA option (refer to [20] for details), and to subsequently correct them with an error correction tool like QUAKE [23].

Another important way to assess a dataset's global quality is to plot the reads' *k-mers distribution*. This can be easily done using Jellyfish [26]. If the genome has been sequenced tens of times, then two peaks are expected: one in correspondence of the expected coverage and one in correspondence of coverage one. *k*-mers composing this second peak are likely to be sequencing errors. As a rule of thumb, a low number of *k*-mers occurring only once suggests that the dataset has a good global quality.

### Contig extension

In the contig extension phase, each read is selected in a loop and used as *seed *in order to create a new contig. Once a *seed *read is selected, the suffix-prefix overlaps with other reads are computed and, if a sufficiently high level of global similarity is reached, they are clustered in a consensus string, which is subsequently used to perform further extensions. The procedure continues while some overlapping reads exist and the consensus string is *highly representative *of the clustered reads. If either one of the previous two conditions is not met, the extension phase stops, the current sequence is returned in output, and the loop continues.

Before the extension phase some parameters are set: the minimum overlap length *L *and the maximum shift Δ: an overlap between the current contig's suffix and the read's prefix is considered only if the overlap length *l *belongs to the interval [*L*, *L *+ Δ].

GapFiller builds a *cluster *every time a contig is to be extended with the overlapping reads. In particular, GapFiller uses only those reads aligning against the contig's suffix with at most *δ *mismatches (where *δ *= *δ*(*l*) is a function of the overlap length *l*) and requires at least *m *reads in order to compute a consensus string. Notice that *b *≤ *L *≤ *l *holds, hence suffix-prefix overlaps might occur with more than *k *= 0 mismatches (see section Definitions).

**Figure 2:**
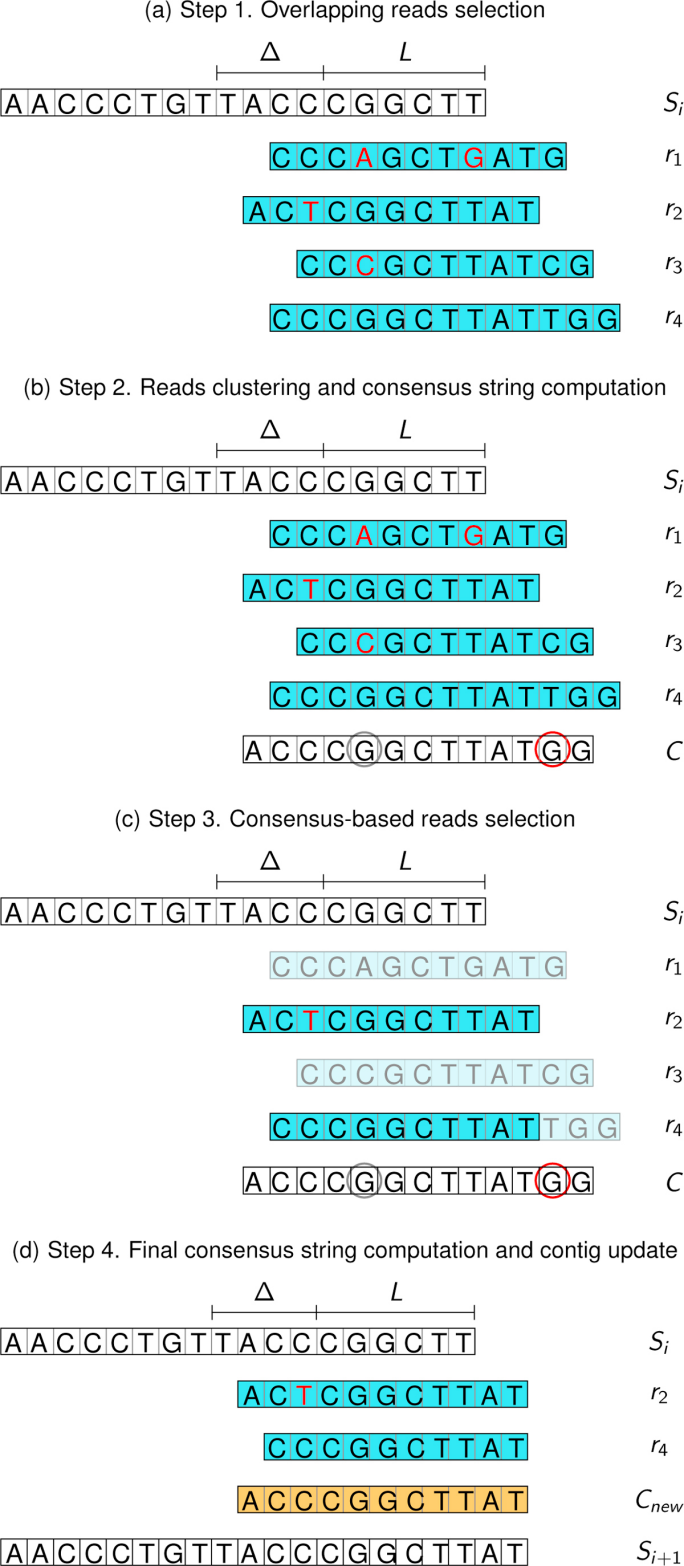
**GapFiller extension phase (an example with *L *= 5, Δ = 4, *δ *= 2, *m *= 2, *T*_1 _= 0.3, *T*_2 _= 0.5)**. (a) The putative overlapping reads, selected by their fingerprint values, are checked for the presence of mismatches and possibly discarded. For each remaining read (say, *r*_1_, *r*_2_, *r*_3_, and *r*_4_), the number of mismatches (highlighted in red) with *S_i_*'s suffix does not exceed *δ *= 2. (b) The consensus string is computed for every position *j *such that either *j *≤ *F *(*C*) or at least *m *= 2 reads are available. The characters rounded in gray and red refer to low-represented and non-represented positions, respectively. In presence of ambiguities (*i.e*., positions in which more than one character with the same representation rate occur) GapFiller chooses the character belonging to the first read encountered, from left to right. (c) Reads with mismatches in correspondence of the low-represented positions are discarded (say, *r*_1 _and *r*_2_), hence they do not contribute to reach the threshold *m *to compute a new consensus string. In our example read *r*_4_'s tail is cut in the non-represented position, regardless on whether it matches the consensus string or not. (d) The reads still alive after Step 3 are used to compute the final consensus string *C_new_*. Since there are 2 ≥ *m *available reads exceeding *S_i_*'s tail, *C_new _*is computed, it is attached to *S_i_*, and the extended contig *S*_*i*+1 _is obtained.

Let ℛ be the set of the input reads for GapFiller and *r*_0 _∈ ℛ be a seed read. At step *i *= 0 the current sequence is initialized with the seed *S*_0 _: = *r*_0_. Denoting by *S_i _*the current contig at the generic *i*-th step of the algorithm, the procedure to build *S*_*i*+1 _is described below:

**Step1 **Reads are selected according to their similarity with the current contig *S*_*i *_(see Figure 2a). At this point, every read overlapping *S*_*i *_for *l *∈ [*L*, *L *+ Δ] characters with at most *δ *mismatches is selected.

**Step2 **The reads are clustered and a consensus string is computed. Every character of the consensus string is assigned a flag indicating how it is representative of the reads from which it is built. More precisely, for every position *j*, GapFiller selects the most occurring character in the considered reads, and the majority consensus string *C *is computed (see Figure 2b). Depending on two parameters *T*_1 _and *T*_2 _such that *T*_1 _<*T*_2_, we say that a position *j *is non-represented, low-represented, or high-represented if the representation rate of the corresponding character in *C *is lower than *T*_1_, lower than *T*_2_, or higher than *T*_2_, respectively.

**Step3 **The reads used to build the consensus *C *are filtered and trimmed, depending on the presence of low-represented and non-represented positions, respectively. The idea is that on low-represented positions we need a minimum percentage of reads matching the consensus string, and that on non-represented positions the extension in considered to be unsafe. Reads differing from *C *in correspondence of low-represented positions are discarded and the remaining ones are also trimmed if a non-represented position occurs (see Figure 2c).

**Step4 **A new consensus string *C_new _*is computed, considering only the reads obtained at Step 3, and possibly the current contig is extended (see Figure 2d). The extension is done only if the number of reads is at least *m *and the consensus *C_new _*exceeds *S_i_*'s right end: in this case, a new contig *S*_*i*+1 _is built and the procedure restarts. Otherwise the algorithm stops and the contig *S_i _*is returned.

The adopted strategy is aimed at either avoiding errors and overcoming the problems arising when GapFiller attempts to cluster reads that are different from each other. In the last part of this section we will discuss in more detail how the algorithm works. The reader who is not interested in the technical formalism might skip this part and move directly to the Subsection Stop criteria.

#### Step 1. Overlapping reads selection

Let us denote with $R\left(S_{i},l\right)$ the set of the putative overlapping reads with respect to the *l*-suffix of *S_i_*, selected by their fingerprint values (see (2), with *y *= |*S*| - *l *+ *x*, for some values of *x *∈ {0, . . ., *l *- *b*}). For every fixed value of *l*, the set of the reads overlapping the *l*-suffix of *S_i _*with at most *δ *mismatches is defined as

(3) $$\hat{R}\left(S_{i},l\right):\left\{r\in R\left(S_{i},l\right):\;d_{H}\left(r\left[0,...,\;l-\;1\right],S_{i}\left[|S_{i}|\;-l,...,\;|S_{i}|-\;1\right]\right)\leq \delta \right\}$$

where *d_H _*:Σ*^l ^*× Σ*^l ^*→ ℝ^+ ^is the Hamming distance. The set of all the overlapping reads will be denoted by

(4) $$\hat{R}\left(S_{i}\right):=\overset{L+\text{Δ}}{\underset{l=L}{⋃}}\hat{R}\left(S_{i},l\right).$$

Given a read $r\in \hat{R}\left(S_{i},l\right),$ we define its starting and ending positions as

(5) $$I\left(r\right):=|S_{i}|-\;l\;F\left(r\right):=I\left(r\right)+|r|-\;1.$$

I(*r*) and *F*(*r*) represent the position of the read *r *with respect to the current contig *S_i_*, therefore we set *I*(*S_i_*) = 0. For instance, in the case depicted in Figure 2, we have $\hat{R}\left(S_{i},8\right)=\left\{r_{1},r_{4}\right\}$ and $\hat{R}\left(S_{i}\right)=\left\{r_{1},r_{2},r_{3},r_{4}\right\}$, *I*(*r*_1_) = 10 and *F*(*r*_1_) = 20.

#### Step 2. Reads clustering and consensus string computation

The subsequent phase consists of the computation of the consensus string obtained from the set of reads $\hat{R}\left(S_{i}\right)$ (see (4)). Notice that, in order to compute reliable extensions, we require the number of reads to be at least *m*, a parameter that may depend on the dataset used. If there exists no *l *such that the *l*-suffix of *S_i _*is covered by at least *m *reads of $\hat{R}\left(S_{i}\right)$, then the procedure stops. Otherwise, the starting and ending positions of the consensus string *C *with respect to *S_i _*can be computed, thanks to (5). In practice, we let the consensus string start from the leftmost reads, *i.e*., those covering the longest suffix of *S_i _*(see, for instance, the read *r*_2 _in Figure 2) and end at the rightmost position in which the number of reads is at least *m*. More precisely, the starting and ending positions of *C *are defined as

$$\begin{array}{ccc} & I\left(C\right):=\;\text{min}\;\left\{I\left(r\right):r\in \hat{R}\left(S_{i}\right)\right\}; & \\ & F\left(C\right):=\;\text{max}\;\left\{F\left(r\right):r\in \hat{R}\left(S_{i}\right)\text{Λ}|\left\{r^{\prime }\in \hat{R}\left(S_{i}\right):F\left(r^{\prime }\right)\geq F\left(r\right)\right\}|\;\geq m\right\}, \end{array}$$

respectively. If *F*(*C*) > |*S_i_*|-1 the procedure continues, otherwise it stops as *S_i _*cannot be further extended. Looking at Figure 1 we have *I*(*C*) = 9 and *F*(*C*) = 21 and the procedure continues since *F*(*C*) >*F *(*S*_*i*+1_) = 17.

The consensus string *C *is then computed by selecting the most represented character at every position. For every *X *∈ Σ and for every *j *= *I*(*C*), . . ., *F*(*C*) we define the number of occurrences of the character *X *in position *j *with respect to *S_i _*as

$$\sigma (X,j):=\;\;|\left\{r\in \hat{R}\left(S_{i}\right):I\left(r\right)\leq j\leq F\left(r\right)\;\text{Λ}\;r\left[j-I\left(r\right)\right]=X\right\}|.$$

The consensus string *C *is defined, for every *j *= *I*(*C*), . . ., *F *(*C*), by setting *C*[*j *- *I*(*C*)] equal to the highest occurring character, *i.e*., the *X *∈ Σ with the highest number of occurrences in position *j*

$$C\left[j-I\left(C\right)\right]:=\text{arg}\;\underset{X\in \sum }{\text{max }}\sigma \left(X,j\right).$$

Loosely speaking, the character selected on a particular position of the consensus string is the most occurring character in the reads on that position; hence *σ*(*C*[*j *- *I*(*C*)], *j*) is the number of occurrences of character *C*[*j *- *I*(*C*)] on position *j*.

#### Step 3. Consensus-based reads selection

As above mentioned, in order to check, on the one hand, whether a read *r *is highly representative of the consensus *C *and, on the other hand, if the extension is "safe", it is important to introduce the notion of *non-represented*, *low-represented*, and *high-represented *characters in the consensus string. We simply define the *representation rate *of the position *j *as

(6) $$\pi \left(j\right):=\frac{\sigma \left(C\left[j-I\left(C\right)\right],j\right)}{\left|\left\{r\in \hat{R}\left(S_{i}\right):I\left(r\right)\leq j\leq F\left(r\right)\right\}\right|}.$$

Hence we fix two threshold values *T*_1 _and *T*_2 _such that 0.25 ≤ *T*_1 _<*T*_2 _< 1 (notice that π (*j*) ∈ [0.25, 1] as |Σ| = 4) and we distinguish three types of positions in the consensus string:

$$\begin{array}{ccc} & j\;\text{is}\;non\text{-}represented\;\Leftrightarrow \pi \left(j\right)\leq T_{1} & \\ & j\;\text{is}\;low\text{-}represented\;\;\Leftrightarrow T_{1}\;<\pi \left(j\right)\leq T_{2}\\ & j\;\text{is}\;high\text{-}represented\Leftrightarrow \pi \left(j\right)>T_{2}. \end{array}$$

The idea is to discard those reads that "differ from *C*" and to cut them out, as there is not sufficiently high evidence that GapFiller is extending correctly. In practice, we do not consider a read *r *if it does not match the consensus string on a low-represented position, *i.e*., *r*[*j -I*(*r*)] ≠ *C*[*j *- *I*(*C*)], for some *j *such that π (*j*) ≤ *T*_2_. Clearly, this applies to non-represented positions as well. Then, we trim every read overlapping any non-represented position of *C*. More precisely, if *j_not _*is the first non-represented position occurring in *r *(*i.e., π *(*j_not_*) ≤ *T*_1_), we consider *r*[0, . . ., *j_not _*- *I*(*r*) - 1] instead of *r*.

After unsafe reads are discarded and the remaining ones are trimmed, a new set of reads, that can be denoted by ${\hat{R}}_{new}\left(S_{i}\right)$, is finally obtained (see Figure 2c). Every read in ${\hat{R}}_{new}\left(S_{i}\right)$ is both matching the consensus string *C *on each low-represented position and not covering any non-represented one. Using this mechanism we take into account only the most representative reads and do not extend the contig with a consensus character when its representation rate is too low.

#### Step 4. Final consensus string computation and contig update

After previous step, the new set of overlapping reads ${\hat{R}}_{new}\left(S_{i}\right)$ is obtained. A new consensus string *C_new _*can be computed as *C *was before. If *F*(*C_new_*) > |*S_i_*| - 1 the extension is performed, the current contig is updated

$$S_{i+1}:=S_{i}\left[0,...,I\left(C_{new}\right)-1\right].C_{new}$$

and the (*i *+ 2)-th extension phase restarts from *S*_*i*+1_.

### Stop criteria

The algorithm described in the previous section may potentially extend a contig for an arbitrarily large number of times, without checking any "global" properties of the current sequence. With our method the extension phase halts if at least one of the following conditions is met: (i) the available overlapping reads for the consensus *C *are less than *m*; (ii) the available overlapping reads for the new consensus *C_new _*are less than *m*; (iii) contig's length exceeds the maximum length; (iv) the seed-mate has been found.

Let *S_i _*be the contig obtained at the *i*-th step, starting from the seed read *r*_0_. Criterion (i) applies when the consensus string *C *does not exceed the current contig. This means that there are no more than *m - *1 overlapping reads, or that they are too short. In such a case, the contig produced is labelled as NO_MORE_EXTENSION.

Criterion (ii) applies when the consensus may have been produced as consequence of the presence of reads belonging to different genomic locations. More precisely, this situation is likely to appear when the consensus extension is "trying" to exit from a repeat. In this case, either too many reads are discarded (due to the presence of low-represented positions) or a significant trimming of them has been performed (as some non-represented positions occur far before the end of the consensus). In such a situation, the extension is halted and the contig is labelled as REPEAT_FOUND.

Criterion (iii) is satisfied as |*S*_*i*+1_| >*L*_max_, where *L*_max _is fixed at the beginning of the algorithm and is usually set to the maximum insert size, plus a tolerance value. In such a situation, we could have been able to continue the extension but, however, we could not find the seed-mate. This suggests that the contig produced may be wrong or, at least, that it contains a high number of unreliable bases. When the maximum allowed length is exceeded, the computation is halted and the contig, labelled as LENGTH_EXCEED, is returned.

Criterion (iv) is used to stop the extension as the mate ${\tilde{r}}_{0}$ of the seed *r*_0 _is found. At the generic *i*-th step, every $p\in \left\{0,...,|s_{i}|-|{\tilde{r}}_{0}|\right\}$ is checked to see whether the following condition is satisfied

(7) $$d_{H}\left(S_{i}\left[p,...,p+|{\tilde{r}}_{0}|-1\right],{\tilde{r}}_{0}\right)\leq M,$$

where *M *is the maximum number of mismatches allowed between ${\tilde{r}}_{0}$ and *S_i_*. Inequality (7) is satisfied if and only if the mate is found in *S_i _*at position *p *with no more than *M *mismatches. This control is performed on-the-fly and hence the positions already checked at the *i*-th step will not be re-checked. The *mate*-*check *criterion is used as a guarantee of correctness of the whole contig. This is in contrast to previous criteria, which are used to detect and prevent errors introduced in the extension phase. From this point of view, criteria (i) and (ii) can be seen as strictly *local*, since no information collected during previous steps is used. In this last case the contig returned is labelled as MATE_FOUND.

### Data structures

In this section we will take a closer look at the data structures designed for our algorithm and at their implementation. GapFiller's *core *is the module working during the extension phase. At this point, we assume that the set ℛ has already been trimmed and possibly filtered.

The basic idea is to pre-compute as much as possible of the useful information on the reads, in order to speed up the computation of the overlaps needed to perform the extension phase. Suppose that GapFiller is working at the (*i*+1)-th step of an extension, with *i *≥ 0, and let *S_i _*be the current contig. When constructing the consensus string *C *(see Figure 2a) we are always interested in obtaining overlaps between *suffixes *of *S_i _*and *prefixes *of reads belonging to ℛ.

**Figure 3:**
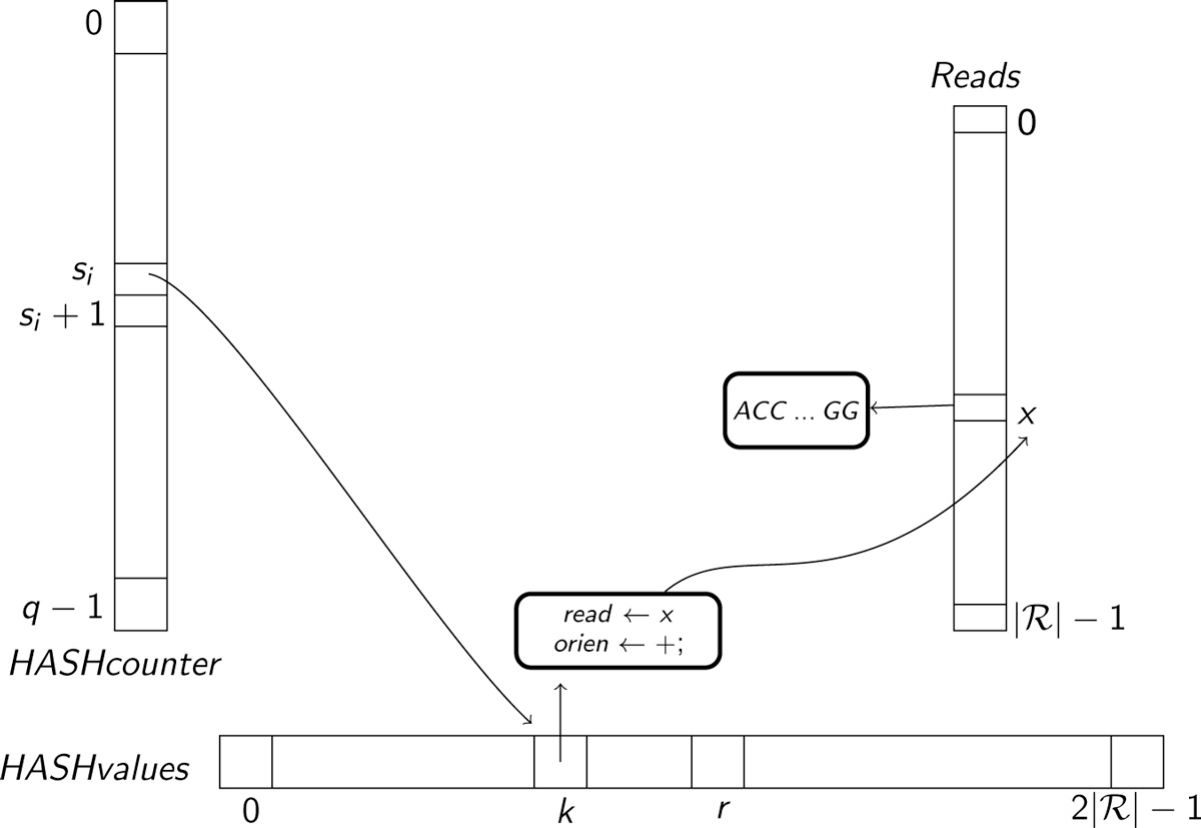
**GapFiller data structure**. The data structure used for GapFiller's implementation is composed of three arrays: *HASHcounter*, whose length depends on the parameter *q *used to compute the fingerprints; *Reads *and *HASHvalues*, whose lengths depend on the number of reads in ℛ. *HASHvalues *is divided in blocks, each of them corresponding to a fingerprint value; each *HASHvalues' *entry contains a pointer to an element of *Reads *and a boolean value indicating whether the read has been reverse-complemented or not.

In order to compute overlaps, GapFiller employs a hashing schema based on the one implemented in rNA [20]; in particular, a data structure similar to the one proposed in [22] is built. A simplified schema of GapFiller's data structure is presented in Figure 3. The basic idea behind GapFiller is the possibility to obtain in a fast and efficient way the set of reads whose prefixes overlap a suffix of the partial contig under construction. Therefore we used the rNA hash function to find reads that are likely to overlap a suffix of *S_i_*; those reads are subsequently checked to see if they actually overlap *S_i _*or not.

Obviously, all the data must be stored in the main memory, thus requiring a careful data structures' engineering. It is clear that, since overlaps between reads and the the current contig can take place on both strands, reads must be stored together with their reverse complement.

With the goal to save as much memory as possible, reads are represented as arrays of integers, so that a base needs 2 bits instead of 8 (A→00, C→01, G→10, T→11). The data structure used to compute overlaps and to construct contigs is built from the reads. Three arrays are used to represent in a compact way the reads stored in ℛ and to compute overlaps among them:

1. *HASHcounter*: it is an array of pointers to *HASHvalues*. In position *i *it stores the first position in *HASHvalues *such that a read *r *or its reverse complement has a prefix whose fingerprint is *i*.

2. *HASHvalues*: each array entry stores the read's location in the array *Reads *together with a boolean value indicating whether the fingerprint has been computed from the original read or from its reverse complement. For this reason the size of *HASHvalues *is twice the number of reads in ℛ;

3. *Reads*: this array stores the reads and other useful informations, like paired read location, paired read order (first or second in a pair), and read status (used, not used, *etc*.).

The overall memory requirement for GapFiller depends on the size of *HASHcounter *and on the number of reads. As for rNA, a reasonable value for *q *is 2^30 ^-1. Such a number guarantees a reduction of the number of false positives (*i.e*., reads reported to align with the contig suffix, even though they do not overlap with it). As far as the number of reads is concerned, we can limit *q*, without loss of generality, to 2^31^: with state-of-the-art Illumina technology, such a number of reads represents approximately a 70× coverage of the human genome. An Illumina read of length 100 bp requires two memory locations in *HASHvalues *of 4 bytes each (31 bits to access array *Reads *and one bit to store the overlap orientation) and one entry in *Reads *of 9 bytes (7 bytes to store the read's numerical representation, one to store the mate position in *Reads*, and one more byte to store several useful informations about read status). In total the amount of memory required is 4*q *+ 2 * 4|ℛ| + 9|ℛ| = 4*q *+ 17|ℛ| bytes.

**Figure 4:**
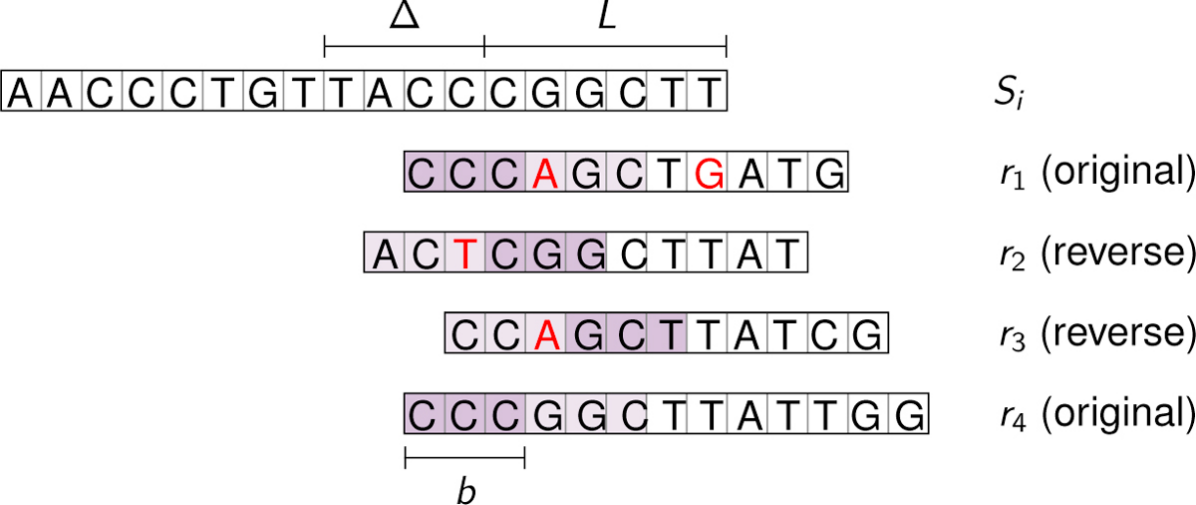
**Reads selection by fingerprint values**. The substring on which the fingerprint is computed must belong to the reads' *L*-prefix in order to be independent on the overlap length *l*. The fingerprint is computed on the leftmost substring of length *b *for original-stranded reads, and on the rightmost *b*-length substring for reverse-complemented ones.

The reads' fingerprint is computed on a precise substring of length *b *(see (2)). As pointed out in section Definitions, the fingerprint of *r *∈ ℛ should be computed on the position *x *such that the (expected) average base quality is as high as possible and the substring *r*[*x*, . . ., *x *+ *b *- 1] falls into the contigs' suffix, independently on the overlap length *l*. For these two reasons, having the Illumina error-profile in mind, we choose *x *= 0 if *r *is considered on its original strand, *x *= *L *- *b *if *r *has been reverse-complemented (see Figure 4).

In order to compute the overlaps between the current contig *S_i _*and the reads, one has to compute the fingerprints of the substrings of length *b *starting from *y*, for every *y *∈ {|*S_i_*| - *L *- Δ, . . ., |*S_i_*| - *L*} if original-stranded reads are searched, and for every *y *∈ {|*S_i _*| - Δ - *b*, . . ., |*S_i_*| - *b*} if reverse-complemented ones are to be extracted. Let us indicate with *s_y _*the fingerprint computed from *S_i_*[*y*, . . ., *y *+ *b *- 1] (see Figure 4). GapFiller uses this number to retrieve reads whose *l*-length prefix (*l *= |*S_i_*| - *y *for original-stranded reads, *l *= |*S_i_*| - *y *+ *L *- *b *for reverse-complemented ones) is likely to match a substring of *S_i _*close to the sequence's end. In particular GapFiller accesses all *HASHvalues *positions between *HASHcounter*[*s_y_*] and *HASHcounter*[*s_y _*+ 1] and, subsequently, accesses *Reads *to identify the set of candidate overlapping sequences ℛ(*S_i_*, *l*) (in Figure 3 GapFiller scans all positions between *k *and *r *- 1 of *HASHvalues*). Finally, the set ℛ(*S_i_*, *l *is used to compute $\hat{R}\left(S_{i},l\right)$, the set of real overlapping reads. This is done by checking all candidate reads singularly. Due to the fact that only a limited number of mismatches is allowed in this phase and that the employed hash function guarantees a low false positive rate, this step is extremely fast.

## Results

GapFiller outputs a set of labelled contigs. The label describes the level of reliability of the sequence, in particular we divide GapFiller's output in two sets: *positive/trusted *contigs are those labelled MATE_FOUND, while *negative/non-trusted *contigs are those labelled NO_MORE_EXTENSION, REPEAT_FOUND, LENGTH_EXCEED. Trusted contigs are those that we consider certified correct and can therefore be used in subsequent analysis. Non-trusted contigs are defined in this way because we were not able to find the seed-mate and hence we have no way to estimate their correctness.

We decided to perform experiments on both simulated and real data. Despite being aware that results on simulated datasets are strongly connected with the ability of read simulators to successfully reproduce realistic error schemata [12], we are also conscious that they are the only way to precisely estimate the reliability of assembled reads. In contrast, experiments on real datasets are necessary in order to test the applicability of our tool.

We simulated NGS experiments on five bacterial genomes, producing four coverages for each of them, in order to show how GapFiller's performances scale at different coverages. Moreover, in order to test correctness, we aligned each output contig against a precise region of the reference, as seed reads' coordinates and orientation are known.

The experiments on real datasets were performed on public data, for which the results obtained by various assemblers are public as well. In this case, we first checked the correctness of GapFiller's output contigs and then used them as input for an assembler for long reads.

### Dataset

The reference genomes used for simulated experiments were downloaded from NCBI website [27] and we used SimSeq, the reads simulator employed in Assemblathon 1 [7], to generate paired reads coverages. More specifically, we performed our experiments on five bacterial genomes (see Table 1). We generated a library constituted by 100 bp-length paired reads, with insert size 600 ± 200 bp, using error profiles provided by SimSeq for reads 1 and 2, respectively. In particular, we obtained 20 simulated datasets generating, for each organism, four paired-ends coverages: 30×, 50×, 70×, and 90×. The reasons behind this choice lie on the fact that, on the one hand, we need at least a 30× coverage in order to provide GapFiller an adequate reads distribution, and, on the other hand, we noticed that coverages equal or higher than 100× do not appreciably increase GapFiller's performances.

**Table 1:** Reference genomes for simulated datasets

Organism	Genome length (bp)	Read length (bp)	Insert size (bp)
*Alcanivorax borkumensis*	3,120,143	100	600
*Alteromonas macleodii*	4,412, 282	100	600
*Bacillus amyloliquefaciens*	3, 980,199	100	600
*Bacillus cereus*	5, 699, 545	100	600
*Bordetella bronchiseptica*	5, 339,179	100	600

The real datasets were dowloaded from GAGE website [28] (see Table 2). Fragment (paired-ends) and short jump (mate-pairs) libraries are available, and corrected data are provided as well. For both datasets, we combined the two libraries in two ways: in a first attempt we ran GapFiller using only reads from the fragment library, while in a second experiment we used both libraries, but we selected seeds from the short jump dataset only, creating in this way contigs of average length 3.5 Kbp.

**Table 2:** Reference genomes and libraries for real datasets (Allpaths error-corrected)

Organism	Genome length (bp)	Library	Avg Read length (bp)	Insert size (bp)	Coverage
*S. aureus*	2, 903, 081	Fragment Short jump	101 96	180 3500	29 × 32 ×
		
*R. sphaeroides*	4, 603, 060	Fragment Short jump	101 101	180 3500	31× 29×

As far as the experiments on real data are concerned, it is important to notice that the datasets provided by GAGE, together with the assembly results described in [9], represent the first available benchmarks that can be used to evaluate new instruments like GapFiller.

Using a specific rNA option, each simulated dataset was filtered to prune and trim reads on the basis of their quality information. For the real datasets, instead, we chose to use the Allpaths error-corrected reads, hence there was no need to trim them.

### Design of experiments

We used simulated data in order to evaluate GapFiller's ability to correctly reconstruct the gap between two paired reads and to assess the reliability of the output classification (NO_MORE_EXTENSION, REPEAT_FOUND, LENGTH_EXCEED, and MATE_FOUND). In particular we used these datasets--easy to build and validate--to explore how coverage affects GapFiller's extension phase. Results on real datasets have been used instead to evaluate GapFiller's potential when its output is used as an input dataset for an assembler for long reads. However, the capability of producing correct contigs is a fundamental feature when GapFiller is used in this context.

GapFiller's performances rely on the choice of three crucial parameters: the minimum overlap length *L*, the slack Δ, and the length *b *of the substring on which the fingerprint is computed. We decided to set *L *= 50 and Δ = 40, as reads' length is approximately 100 bp for every library used for the experiments. The value of *b *identifies the length of a substring on which we (almost always) require an exact matching between read and contig (see Figure 4), due to the fact that the employed hash function has a low false-positives rate (see (2)). We set *b *= 20 because we observed that a greater value of *b *(*i.e*., close to *L*) dramatically prevents GapFiller to find even few-mismatch-affected overlaps.

The parameters *T*_1 _and *T*_2_, necessary to discern among high/low/non-represented positions in the consensus string (see Subsection Implementation-Contig extension), are set to *T*_1 _= 0.6 and *T*_2 _= 0.9. Recall that when a position in the consensus string has a representation rate lower than *T*_1_, all the reads are trimmed on that position; instead, if the representation rate is lower than *T*_2_, only the reads not matching the consensus string are dropped. The value of *m*, the minimum number of reads required in order to compute the consensus string, has always been set to 2. We chose not to let *m *depend upon coverage, since the number of reads after Step 3 strongly depends on the parameters used (say, *T*_1 _and *T*_2_).

We set the maximum length of a contig to be much greater than the expected mean insert size, *i.e*., 1800 bp for simulated data, 550 bp and 4500 bp for GAGE fragment and short jump libraries, respectively (see also Table 1 and Table 2).

We allowed for the presence of mismatches when looking for the seed-mate in the contig being constructed with parameter *M*. In all the performed experiments we set *M *= 10 (*i.e*., approximately 10% of the reads' length). This choice is justified by two reasons: the first one lies in the fact that the data simulated with SimSeq have a quite high amount of low-quality bases even far from the rightmost positions within the reads; the second one is that, on real datasets, lower values of *M *(*e.g*., 5 or 2) do not increase output quality. The value of *δ*, representing the maximum number of mismatches allowed when computing overlaps, depends on the overlap length *l *and was set to *Ml */ |*r*|, where |*r*| is the average read length.

### Analysis

The post-processing phase of GapFiller's output is aimed at both quantitative and qualitative analysis. The first is focused on evaluating the amount of trusted contigs our tool is able to produce, the second on results' validation. The main goal is to compare the performances on different input datasets and coverages.

Due to their nature, experiments on simulated data allow to precisely estimate correctness by aligning a contig in the exact place where it is supposed to occur in the reference genome. More precisely, we used the Smith-Waterman alignment algorithm [29], assigning a score of 1 to a match, -1 to a mismatch, and -2 to an indel. For instance, let us consider a contig *S *generated by extending a seed read *r*, and suppose that *r *has been extracted from the genome *G *at position *x*, on the forward strand. To test its correctness, *S *is aligned against *G*[*x*, *x *+ |*S*| + *g *- 1], where *g *is the maximum number of allowed indels, depending on a user-defined threshold for the alignment score. We say that *S *is *correctly aligned *if and only if the ratio between the best alignment score of *S *against *G*[*x*, *x *+ |*S*| + *g *- 1] and |*S*| is at least 0.95 (for instance, we allow up to 5 mismatches, 1 indel and 1 mismatch, or 3 indels every 200 bp, on average). For this particular choice of the alignment score, *g *is fixed to be ⌈3|*S*|/200⌉.

Alignments performed in this way allowed us to divide contigs in four subsets: *true *and *false *positives and *true *and *false *negatives, depending on the contigs classification and correctness (see Table 3). This gave us the possibility not only to estimate the percentage of correctly reconstructed contigs, but also to evaluate GapFiller's ability to discern between trusted and not trusted ones.

**Table 3:** Contigs post-processing classification

	Aligned	Unaligned
**Trusted**	True Positive (TP)	False Positive (FP)

**Not trusted**	False Negative (FN)	True Negative (TN)

When using a real dataset reads provenance is unknown, so in this case we tested output correctness by aligning the contigs against the reference genome using BLAST. We set the percentage of identity to be at least 95% and the hit length to be 100% of contig's length, in order to accept an alignment. In real cases it is interesting to extract two pieces of information from alignments: the number of (trusted) contigs that correctly align against the reference, as in the simulated case, and the coverage profile, as it is useful in order to estimate the percentage of genome reconstructed by GapFiller (see Table 4).

**Table 4:** Validation of GapFiller's output on GAGE datasets

Organism	Library	Avg contig length (bp)	Aligned contigs	Aligned length	Genome cov
*S. aureus*	Fragment S.j. + fragment	182 3648	99.48% 98.74%	99.47% 98.76%	98.12% 95.00%

*R. sphaeroides*	Fragment S.j. + fragment	188 3736	99.91% 98.20%	99.92% 98.22%	98.65% 74.12%

The experiments performed with both short jump (s.j.) and fragment libraries are done by picking the seeds from the short jump library only. We state that a contig is aligned against the reference if the alignment is a single hit covering 100% of contig's length and the percentage of identity is at least 95%. The statistics are computed on trusted contigs.

Thanks to the presence of *theoretical optimal *assemblies for the two real datasets (see [9]) we evaluated the performances of GapFiller with respect to other assemblers. In particular, we extracted a set of contigs corresponding to a fixed coverage (10× for *Staphylococcus aureus *and 15× for *Rhodobacter sphaeroides *datasets, respectively) and assembled it with PHRAP [30], a well known Overlap-Layout-Consensus assembler. We produced a set of statistics representing the correctness of our assembly using the same scripts used in [9] and available for download at [28].

## Discussion

All the experiments were performed on a 8CPU (2500GHz) and 32GB RAM machine. All of them required no more than ~ 5.4GB RAM memory. See Table 5 for the time requirements and for the output coverage produced for each experiment.

**Table 5:** GapFiller performances on both simulated and real datasets

Organism	Dataset	Output coverage	Time
*A. borkumensis*	30×	80×	25' 45"
	50×	141×	53' 14"
	70×	199×	1 h 30' 23"
	90×	279×	2 h 01' 03"

*A. macleodii*	30×	83×	30' 55"
	50×	146×	1 h 12' 12"
	70×	203×	2 h 05' 36"
	90×	262×	3 h 12' 36"

*B. amyloliquefaciens*	30×	87×	26' 40"
	50×	154×	1 h 01' 24
	70×	216×	1 h 47' 51"
	90×	278×	2 h 44' 52"

*B.cereus*	30×	86×	35' 54
	50×	151×	1 h 20' 50"
	70×	213×	2 h 21' 28
	90×	274×	3 h 36' 37"

*B. bronchiseptica*	30×	87×	35' 27"
	50×	153×	1 h 19' 34"
	70×	215×	2 h 19' 01"
	90×	276×	3 h 35' 01"

*S. aureus*	Fragment	26×	08' 25"
	Short jump + fragment	517×	3 h 34' 01"

*R. sphaeroides*	Fragment	28×	08' 43"
	Short jump + fragment	230×	5 h 27' 21"

**Figure 5:**
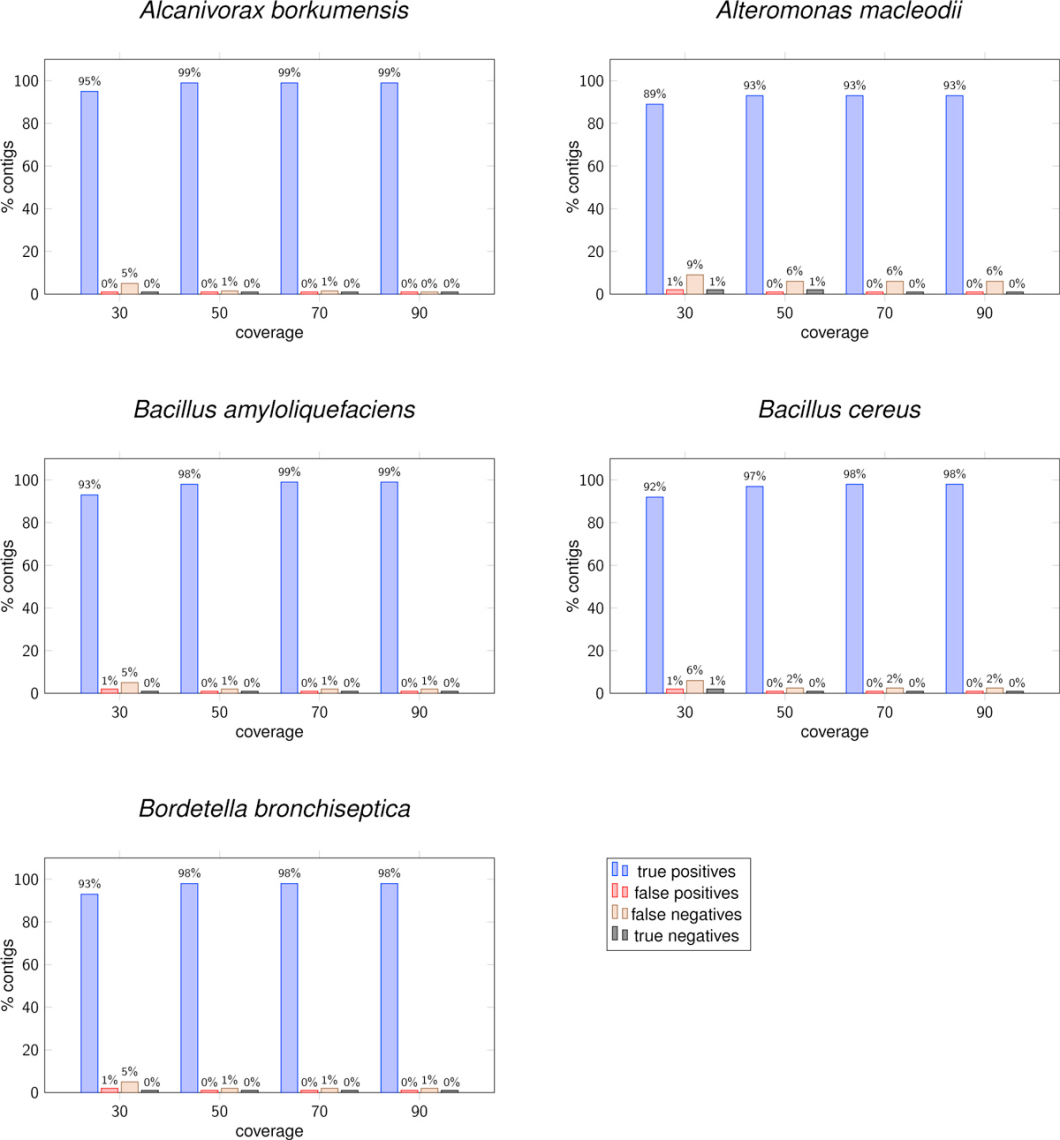
**Results on 5 simulated datasets**. The five histograms represent, for each dataset, the true positives, false positives, false negatives, and true negatives rates for different input coverages. In order to decide if a (positive or negative) contig is either true or false, it is aligned against the reference on the exact positions in which it is supposed to occur.

Experiments performed on simulated datasets show how GapFiller's performances improve as coverage increases (see Figure 5 and Table 5). From the histograms in Figure 5 we can clearly appreciate how the number of true positives (see Table 3) increases with coverage, reaching an average value of 99% when coverage is above 50×. In a specular way, we can see that the number of false negatives decreases as coverage increases. Table 5 shows how a higher input coverage allows us to produce a higher output coverage composed by trusted reads.

The simulated datasets allowed us to show how GapFiller is able not only to correctly reconstruct the gap between paired reads, but also to correctly flag the generated contigs as trusted (*i.e*., MATE_FOUND) and non-trusted (all other cases). Going into more detail, we observed that the majority of non-trusted contigs are labelled NO_MORE_EXTENSION, meaning that GapFiller stops a contig extension depending on some input dataset features (low covered regions and/or error-affected reads). Another possible scenario is the one in which GapFiller computes a wrong consensus without recognizing it.

Another important result obtained from these datasets is that the percentage of uncovered bases is negligible, being strictly less than 0.1% even with low input coverages (*e.g*., 30×).

On the basis of the results obtained on simulated data, we tested GapFiller on real data. We decided to use two datasets provided by GAGE [9]. We opted for these data because they represent state-of-the-art Illumina sequences, they are freely available, and they come with a reference sequence, a set of assemblies obtained with state-of-the-art assemblies, and with a set of evaluation scripts.

Table 4 shows GapFiller's results on *Staphylococcus aureus *and *Rhodobacter sphaeroides *datasets. For both of them we run GapFiller twice: a first time using only reads from fragment library and a second time using reads from short jump library as seeds and reads from both libraries to close the gap. From Table 4 we can also see that, in both situations, GapFiller is able to reconstruct the insert with the expected size; moreover the amount of aligned trusted contigs is comparable to that obtained when simulated datasets are used as input. The percentage of reconstructed genome is extremely high in *S*. *aureus *(for both experiments) and *R*. *sphaeroides *when fragment library is used alone. When reads from short jump library are used as seeds, instead, there is almost 26% of reference missing. This could have been caused either by a bias in the library (non-uniform mate-pairs distribution) or by the presence of difficult-to-assemble areas larger than the insert size.

In order to proof GapFiller's capabilities when used on real data, we extracted a random 10× coverage from the set of *S*. *aureus *output contigs (in particular from those obtained using short jump reads as seeds) and a random 15× coverage from *R*. *sphaeroides *output contigs (10× and 5× from those obtained using seeds from fragment and short jump libraries, respectively). Both coverages have been assembled with PHRAP with default parameters and the results have been compared to the ones presented in GAGE [9]. It is worth noting that the assemblies presented in GAGE should be considered *the best *achievable assemblies with the employed tools. In order to obtain a comparison as fair as possible we employed the same scripts used by Salzberg and colleagues in [9]. It is important to say that the presence of a reference sequence for both the assembled genomes allows us to compute the real number of errors and mis-assemblies.

Tables 6 and 7 show the most important statistics obtained in the validation phase. For what concerns *S*. *aureus *assemblies, we can see that our assembly has a connectivity level (number of contigs and N50) higher than that of many other widely used assemblers (*e.g*., Velvet), moreover the number of small contigs (chaffs), and the number of wrongly assembled repeats (duplications and compressions) is always comparable and often better than the other assemblies (all percentages in Tables 6 and 7 are expressed as a pecentage of true genome size). The most important columns, however, are the last four, showing the number of errors (the ideal assembler should have 0 everywhere). GapFiller+PHRAP not only is one of the assemblies with the fewest number of indels, but is also the one having less relocations (3) and inversions (0). These latest two types of errors are the most dangerous ones, due to the fact that they are the result of merging two completely different genome areas.

**Table 6:** GAGE comparison statistics on Staphylococcus aureus contigs

Assembler	#Ctg	NG50	Chaff %	Dupl %	Comp %	Indels ≤ 5 bp	Indels > 5 bp	Inv	Rel
ABySS	301	29198	6.71	23.06	0.98	20	9	3	2
Allpaths-LG	59	96740	0.03	0.03	1.26	4	12	0	4
Bambus2	108	50192	0.00	0.01	1.27	56	164	2	11
MSR-CA	93	59152	0.02	0.71	0.88	23	10	6	7
**GapFiller+PHRAP**	**90**	**42398**	**0.00**	**0.28**	**1.07**	**12**	**4**	**0**	**3**
SGA	1253	4005	21.34	0.01	1.26	2	2	1	3
SOAPdenovo	106	288184	0.35	1.42	1.39	25	31	1	16
Velvet	161	48440	0.46	0.14	1.31	6	14	5	9

**Table 7:** GAGE comparison statistics on Rhodobacter sphaeroides contigs

Assembler	#Ctg	NG50	Chaff %	Dupl %	Comp %	Indels ≤ 5 bp	Indels > 5 bp	Inv	Rel
ABySS	1916	5872	1.67	10.07	0.49	278	34	2	17
Allpaths-LG	203	42455	0.01	0.38	0.33	150	37	0	6
Bambus2	176	93198	0.00	0.00	0.25	149	363	0	5
CABOG	321	20211	0.00	0.12	0.71	145	24	1	9
MSR-CA	394	22128	0.02	1.05	0.53	179	32	1	8
**GapFiller+PHRAP**	**1584**	**7809**	**0.12**	**0.49**	**0.76**	**158**	**14**	**2**	**7**
SGA	3073	2284	3.49	0.05	0.98	114	5	0	5
SOAPdenovo	204	131681	0.44	1.07	0.54	155	406	0	8
Velvet	583	15665	0.55	0.29	0.96	148	27	0	8

Results showed in Table 7 for *R*. *sphaeroides *are similar: this time GapFiller+PHRAP has a lower connectivity level (however greater than SGA and ABySS, two widely used assemblers). Also in this case our assembly is not seriously affected by indels (opposite to SOAPdenovo that has more than 550 indels). Concerning inversions and relocations, GapFiller + PHRAP's performances are comparable to that of the other assemblers.

## Conclusion

GapFiller is a *local assembler *based on a hashing technique. Indeed, on the one hand, it boosts the extension phase by reducing the search space and hence allows an exact computation of overlaps, and, on the other hand, it allows to store in an efficient and compact way all the needed information.

GapFiller is a tool able to provide *certified *contigs, in the sense that those labelled "trusted" are (almost always) correct. This statement is sustained by various simulated experiments, as well as by two real ones. GapFiller does not try and does not aim at assembling a genome but, instead, it aims at providing as output a set of Sanger-like-length reads certified correct. In a *de novo *assembly project, GapFiller can be used in two modalities. It can realize a preprocessing step, as the produced trusted contigs can be used as input *meta-reads *for an assembler for long reads; as an opposite strategy, it can be used to join the contigs produced by a *de novo *assembler in a scaffolding-like phase or to (partially) assemble structural variations within an NGS resequencing project.

In this paper we proved the effectiveness of the first application. We showed how the Sanger-like-long reads can be used to feed another assembler (PHRAP [30] in our case, but many other solutions are possible) in order to obtain a standard assembly. This assembly is similar and often better than assemblies generated by state-of-the-art assemblers. In order to proof this we compare the results of our tool with the ones recently obtained by GAGE.

GapFiller's strength lies, on the one hand, in the ability to produce an output that does not need validation, and, on the other hand, in being a *local *assembler, making it useful when studying limited regions of a genome.

GapFiller's applications to structural variations analysis include indels detection and validation; in particular, it can be used to assemble insertions occurred in a sequenced organism, with respect to a reference genome. It is of primary importance to notice how, while there is a large number of tools able to *identify *structural variations, so far there is no widely accepted strategy to *reconstruct *structural variations in re-sequencing projects. We believe that the localized GapFiller strategy can be used in order to "fill this gap" and move several approaches from identification to reconstruction.

## Availability and requirements

GapFiller can be freely downloaded from http://users.dimi.uniud.it/~francesco.vezzi/software.php. It has been tested on Linux Operating systems only (Ubuntu and Centos distributions). It has been written in C++.

## Abbreviations

NGS: Next Generation Sequencing.

## Competing interests

The authors declare that they have no competing interests.

## Authors' contributions

FN, FV, and AP equally contributed to the idea and equally contributed to the design of the experiments. FN and FV developed the tools and FN performed the experiments. FN, FV, and AP wrote the paper.

## Appendix

SimSeq can be freely downloaded from https://github.com/jstjohn/SimSeq.

Command lines for read simulation:

java -jar -Xmx2048m SimSeq.jar -1 100 -2 100 --error hiseq_mito_default_bwa_mapping_mq10_1.txt

--error2 hiseq_mito_default_bwa_mapping_mq10_2.txt --insert_size 600 --insert_stdev 200

--read_number PAIR_NUMBER --reference reference.fasta -o output.sam;

java -jar SamToFastq.jar INPUT=output.sam FASTQ=reads_1.fastq SECOND_END_FASTQ=reads_2.fastq

INCLUDE_NON_PF_READS=true VALIDATION_STRINGENCY=SILENT

KmerCounter can be freely downloaded from its git repository git clone

http://git://git.code.sf.net/p/kmercounter/code kmercounter-code. Command line for KmerCounter:

./kmers_count --input reads_1.fastq --input reads_2.fastq --threads NUM_THREADS

--output 16mer_profile.txt (--mark-reads READ_NAME)

Command line for GapFiller:

./IGAassembler --k 15 --output output.fasta --statistics output.stat --overlap 50 --slack 40

--short-1 seed_reads.fasta --short-2 seed_mates.fasta (--short-1 reads1.fasta

--short-2 reads2.fasta) --short-ins AVG_INSERT_SIZE --short-var INSERT_SIZE_ST_DEV

--read-length AVG_READ_LENGTH --global-mismatch 10 --extThr 2 --limit NUM_SEEDS_TO_EXTEND

--no-read-cycle --max-length MAX_CTG_LENGTH
